# The catalytic domains of *Streptococcus mutans* glucosyltransferases: a structural analysis

**DOI:** 10.1107/S2053230X23003199

**Published:** 2023-05-05

**Authors:** Norbert Schormann, Manisha Patel, Luke Thannickal, Sangeetha Purushotham, Ren Wu, Joshua L. Mieher, Hui Wu, Champion Deivanayagam

**Affiliations:** a Department of Biochemistry and Molecular Genetics, University of Alabama at Birmingham, Birmingham, AL 35294, USA; b School of Dentistry, Oregon Health and Science University, Portland, OR 97201, USA; Station Biologique de Roscoff, France

**Keywords:** glucansucrases, glycosyltransferases, catalytic domains, soluble and insoluble glucans, GtfB, GtfC, GtfD, dental plaque, *Streptococcus mutans*, SRCR_1_ binding

## Abstract

*Streptococcus mutans*, found in the human oral cavity, is a significant contributor to the pathogenesis of dental caries. This bacterium expresses three genetically distinct types of glucosyltransferases named GtfB (GTF-I), GtfC (GTF-SI) and GtfD (GTF-S) that play critical roles in the development of dental plaque. The catalytic domains of GtfB, GtfC and GtfD contain conserved active-site residues for the overall enzymatic activity that relate to hydrolytic glycosidic cleavage of sucrose to glucose and fructose, release of fructose and generation of a glycosyl-enzyme intermediate in the reducing end. In a subsequent transglycosylation step, the glucosyl moiety is transferred to the nonreducing end of an acceptor to form a growing glucan polymer chain made up of glucose molecules. It has been proposed that both sucrose breakdown and glucan synthesis occur in the same active site of the catalytic domain, although the active site does not appear to be large enough to accommodate both functions. These three enzymes belong to glycoside hydrolase family 70 (GH70), which shows homology to glycoside hydrolase family 13 (GH13). GtfC synthesizes both soluble and insoluble glucans (α-1,3 and α-1,6 glycosidic linkages), while GtfB and GtfD synthesize only insoluble or soluble glucans, respectively. Here, crystal structures of the catalytic domains of GtfB and GtfD are reported. These structures are compared with previously determined structures of the catalytic domain of GtfC. With this work, apo structures and inhibitor-complex structures with acarbose are now available for the catalytic domains of GtfC and GtfB. The structure of GtfC with maltose allows further identification and comparison of active-site residues. A model of sucrose binding to GtfB is also included. The new structure of the catalytic domain of GtfD affords a structural comparison of the three *S. mutans* glycosyltransferases. Unfortunately, the catalytic domain of GtfD is not complete since crystallization resulted in the structure of a truncated protein lacking approximately 200 N-terminal residues of domain IV.

## Abstract 2

Apo and acarbose-complex structures of the catalytic domain of GtfB from *Streptococcus mutans* and the truncated structure of the catalytic domain of GtfD are described.

## 1. Introduction

*Streptococcus mutans*, a known etiological agent in the pathogenesis of dental caries, expresses three genetically distinct types of glucosyltransferases (Gtfs; also known as glucansucrases): GtfB (GTF-I), GtfC (GTF-SI) and GtfD (GTF-S). GtfC synthesizes both soluble and insoluble glucans (α-1,3 and α-1,6 glycosidic linkages), while GtfB and GtfD only synthesize insoluble glucans (mostly α-1,3 linkages) and soluble glucans (mostly α-1,6 linkages), respectively. All three enzymes belong to glycoside hydrolase family 70 (GH70), which cleaves the glycosidic bond between the glucose and fructose moieties in sucrose during catalysis. While fructose is released, the enzymatically produced glucose molecules are sequentially fused into a growing glucan chain and, depending on whether a 1,3- or 1,6-linkage is formed, the resultant product is either an insoluble or a soluble glucan, respectively. Based on homology to the GH13 family, Robyt and coworkers proposed that both sucrose breakdown and glucan synthesis occur within the same active site, although the size of the active site calls this into question (Robyt, 1995 ▸; Robyt *et al.*, 2008 ▸). It needs to be noted that the active site in the GH13 family is larger than the active site observed in the GH70 family.

Dental plaque is a biofilm consisting of a group of microorganisms embedded in an extracellular polysaccharide (EPS) matrix which is attached to the surface of the tooth. The primary source of EPS in dental plaque are products of the interaction of glucosyltransferases and fructosyltransferases with sucrose and starch hydrolysate (Vacca-Smith *et al.*, 1996 ▸). The establishment of this matrix is a result of the simultaneous synthesis of glucans by the surface-adsorbed GtfB and GtfC enzymes. Therefore, these Gtfs influence the microbial colon­ization of tooth surfaces. Gtfs are predominantly observed within the oral cavity, and the presence of active *S. mutans* Gtfs within the dental pellicle facilitates the formation of glucans *in situ*, thus enabling other oral microorganisms to adhere and colonize. Among these three Gtfs, GtfB binds with higher avidity to oral microorganisms compared with GtfC or GtfD, thereby promoting cell clustering and microbial co­hesion within the plaque biofilms (Zhang *et al.*, 2021 ▸). GtfC, on the other hand, has the greatest affinity for saliva-coated hydroxyapatite, while GtfD acts as a primer of GtfB (Zhang *et al.*, 2021 ▸).

Functionally, all three Gtfs are known to play a critical role in the development of dental plaque. Current knowledge shows that GtfD is dependent on the acceptor (for instance dextran) for glucan synthesis; on the other hand, GtfB and GtfC are independent of the exogenous glucan acceptor, although their enzymatic activity is enhanced in the presence of dextran. A detailed mutational analysis of all three *S. mutans* Gtfs, GtfB, GtfC and GtfD, has identified multiple key residues that are important for sucrose hydrolysis (Shimamura *et al.*, 1994 ▸; Vacca-Smith *et al.*, 1996 ▸; Chia *et al.*, 1998 ▸). The crystal structure of the catalytic domain of GtfC showed the presence of the Ca^2+^-binding residues Asp437, Asp959, Glu431 and Asn481 (Ito *et al.*, 2011 ▸). Mutation of one of the two Ca^2+^-binding aspartate residues in GtfB (Asp411) and GtfC (Asp437) affected the sucrase activity, indicating that structural stability is provided by the Ca^2+^ (Shimamura *et al.*, 1994 ▸; Chia *et al.*, 1998 ▸). In addition, another aspartate residue (Asp413 in GtfB, Asp439 in GtfC and Asp427 in GtfD) adjacent to the previous aspartate but not part of the Ca^2+^-binding site also proved to be important. Mutation of these aspartate residues to asparagine resulted in loss of enzymatic activity (Shimamura *et al.*, 1994 ▸; Chia *et al.*, 1998 ▸). The catalytic domains of GtfB, GtfC and GtfD are capable of the hydrolysis of sucrose to glucose (glucosyl-enzyme intermediate) and fructose, although they are not able to produce extensive amounts of glucan polymers without the inclusion of domain V that contains the glucan-binding repeats (Kato & Kuramitsu, 1990 ▸; Lis *et al.*, 1995 ▸).

The sucrase active site has been described in multiple studies of Gtfs not only in *S. mutans* but also in *Leuconostoc citreum* and *Lactobacillus reuteri* (Wangpaiboon *et al.*, 2018 ▸, 2020 ▸; Vujičić-Žagar *et al.*, 2010 ▸). In the crystal structure of GtfC, this site is defined by the nomenclature (−*n*, non­reducing end; +*n*, reducing end), and the co-crystal structure with acarbose places the nonreducing end of sucrose (−*n*; glucose) at the active site that consists of Asp437 (nucleophile), Glu431 (acid–base catalyst) and Asp959 (transition-state stabilizer). Mutational analysis of these residues confirmed that this site possesses sucrase activity. In an extensive comparative analysis of the active-site residues in GtfB, GtfC and GtfD, one residue was found to decide the sugar connectivity in GtfD. This residue, Thr589, was mutated to an aspartate in accordance with GtfC (Asp593) and GtfB (Asp567), which resulted in the production of insoluble glucans (Shimamura *et al.*, 1994 ▸; Chia *et al.*, 1998 ▸).

Thus far, only the structure of the catalytic domain of GtfC has been elucidated (Ito *et al.*, 2010 ▸, 2011 ▸). In the current work, we set out to characterize the catalytic domain structures of GtfB and GtfD, with the aim of identifying structural and functional differences between these three distinct Gtfs produced by *S. mutans*. More importantly, we have now co-crystallized the GtfB with acarbose, which highlights differences in the interactions with ligands in these distinct Gtfs.

## 2. Materials and methods

### 2.1. Cloning of the catalytic domains of GtfB and GtfD

Cloning of the *S. mutans* (UA159) GtfB catalytic domain spanning residues 191–1051 (UniProt sequence P08987) into a pET-23d vector (Novagen) has previously been described (Mieher *et al.*, 2019 ▸). The *S. mutans* (UA159) catalytic domain of GtfD spanning residues 248–1091 (UniProt sequence P49331) was cloned into a pET-21a vector (Novagen). The forward and reverse primers are shown in Table 1 ▸. Both constructs contain a C-terminal histidine tag for easy affinity purification.

### 2.2. Expression and purification

The catalytic domains of *S. mutans* GtfB and GtfD were expressed in *Escherichia coli* BL21(DE3) cells harboring pET-23d or pET-21a vectors that contain the gene inserts representing residues 191–1051 of GtfB and 248–1091 of GtfD, respectively.

Purification of the catalytic domain of GtfB has previously been described (Mieher *et al.*, 2019 ▸). Briefly, after sonicating the cells in binding buffer (50 m*M* Tris pH 7.9, 500 m*M* NaCl) and using the C-terminal His tag, the protein was bound to a HisTrap FF column (GE Healthcare) and washed with 20 column volumes (CV) of the same buffer to remove unbound protein and then with 10 CV of binding buffer supplemented with 50 m*M* imidazole to remove nonspecifically bound protein(s). After this step, a gradient of 50–300 m*M* imidazole was performed over 15 CV to elute the catalytic domain of GtfB. After confirming their identity by SDS–PAGE, fractions with a predominate band at about 100 kDa were pooled together and dialyzed overnight in ion-exchange binding buffer (20 m*M* Tris pH 7.5, 50 m*M* NaCl). They were subsequently loaded onto a Mono Q 10/100 column (GE Healthcare) and eluted using an NaCl gradient. The purest fractions identified by SDS–PAGE were pooled and subjected to size-exclusion chromatography on a Superdex 200 26/60 column (GE Healthcare). Based on SDS–PAGE (a single band at about 100 kDa), the purity was assessed to be greater than 99%. The initially obtained GtfB protein showed proteolysis during crystallization trials at room temperature, which we were later able to remedy by inclusion of the Roche protease-inhibitor cocktail as well as AEBSF (AmericanBio) after the final purification step. The protein was concentrated to 15.8 mg ml^−1^ using an Amicon stirred-cell concentrator under a nitrogen pressure of 0.379 MPa.

The catalytic domain of *S. mutans* GtfD was purified in a similar fashion and concentrated to 13.4 mg ml^−1^. SDS–PAGE showed a single band at about 100 kDa that subsequently showed no proteolysis when samples that had been frozen at −80°C were analyzed. Under room-temperature conditions limited proteolysis was detected that resulted in a truncated version during crystallization trials. In this case, addition of the Roche protease-inhibitor cocktail as well as AEBSF did not prevent proteolysis during crystallization.

The sucrase activity of the purified catalytic domains of GtfB and GtfD was verified by the 3,5-dinitrosalicylic acid assay for reducing sugars using the Nelson–Somogyi method described in the literature (Gusakov *et al.*, 2011 ▸). No attempt was made to check for glucan synthesis, since it has been reported previously that catalytic domains lacking the glucan-binding repeats of domain V do not produce extended glucan polymers (Kato & Kuramitsu, 1990 ▸; Lis *et al.*, 1995 ▸).

### 2.3. Crystallization and data collection

#### 2.3.1. GtfB-CD

The truncated form (residues 423–932; PDB entry 8fkl) of the catalytic domain of GtfB (GtfB-CD) crystallized in multiple precipitant conditions including PEG 3350, PEG 6000, PEG 8000 and PEG 10 000 from four different high-throughput screens (JCSG Core Suite I from Qiagen and LMB, ProPlex and Morpheus from Molecular Dimensions). These crystals were very ‘sturdy’ and block-like despite having a solvent content of about 60%. The best crystals, which were obtained in LMB condition G11 (20% PEG 8000, 0.1 *M* CAPS pH 9.0, 0.2 *M* MgCl_2_), diffracted to 1.48 Å resolution.

Crystals of intact GtfB-CD (PDB entry 8fk4) co-crystallized with 0.8 m*M* acarbose (10:1 molar ratio of inhibitor to protein) grew in condition A3 (2 *M* ammonium sulfate, 0.1 *M* bis-Tris pH 5.5) of Index (Hampton Research) using our Gryphon crystallization robot (Art Robbins Instruments) in a 96-well Corning 3350 plate (sitting-drop vapor-diffusion technique). These crystals were very soft and fragile due to the high solvent content of greater than 70% despite having eight molecules in the asymmetric unit. The crystals belonged to the orthorhombic crystal system and diffracted to a resolution of 3.25 Å (Mieher *et al.*, 2019 ▸). They were structurally homologous (space group and unit-cell parameters) to the three previously reported GtfC structures (Ito *et al.*, 2011 ▸).

The hanging-drop vapor-diffusion technique in a Linbro plate using the same crystallization condition (Index A3) with various drop ratios (1:1, 2:1 and 1:2) and drop sizes (2, 3 and 4 µl) reliably produced large enough (0.1–0.2 mm) block-like apo crystals of GtfB-CD (PDB entry 8fj9). These crystals adopted the tetragonal crystal system and displayed markedly improved diffraction (resolution of 2.50 Å). Some of the apo crystals were soaked with acarbose (final concentration 1–2 m*M*) to obtain a protein–inhibitor complex (PDB entry 8fjc).

#### 2.3.2. GtfD-CD

Crystals of the catalytic domain of GtfD (GtfD-CD; PDB entry 8fn5) grew in multiple screen conditions of LMB (Molecular Dimensions) and Index (Hampton Research), and crystals obtained in LMB condition C9 (26% PEG 2000 MME, 0.1 *M* bis-Tris pH 5.8) diffracted to 1.92 Å resolution. Prediction of the Matthews coefficient based on the unit-cell parameters indicated a truncated protein (although the purified protein and protein frozen at −80°C showed an SDS–PAGE band at about 100 kDa) that underwent proteolysis during crystallization trials at room temperature. This was later confirmed after molecular replace­ment and refinement (see Section 2.4). This GtfD-CD structure lacks the N-terminal 200 residues of domain IV, which includes the residues that coordinate the conserved Ca^2+^ ion.

#### 2.3.3. Data collection

Diffraction data for all five structures (four GtfB-CD structures and one GtfD-CD structure) were collected at 100 K using a Dectris EIGER 16M hybrid pixel detector on the SER-CAT 22-ID beamline at the Advanced Photon Source (APS), Chicago, USA. Data were collected at a wavelength of 1 Å with a crystal-to-detector distance of 300 mm, a rotation of 0.25° per frame (2θ) and an exposure of 0.25 s, covering 200° of reciprocal space (800 frames in total). For cryoprotection of the GtfB-CD crystals 20% ethylene glycol was added to the crystallization buffer, while no cryosolution was required for GtfD-CD. The collected data were processed using *XDS* (Kabsch, 2010*a* ▸,*b* ▸) for initial indexing, merging and scaling; this was followed by optimization using *AIMLESS* (Evans, 2011 ▸) in *CCP*4 (Winn *et al.*, 2011 ▸). Because of the overall high redundancy of the collected reflections, selected batches of reflections with unsatisfactory quality (for example high *R*_merge_ values) were removed to obtain a high-quality data set. Data-collection statistics are shown in Table 2 ▸.

### 2.4. Structure determination and refinement

The structures of GtfB-CD and GtfD-CD were solved by molecular replacement with *Phaser* (McCoy *et al.*, 2007 ▸) using models of GtfB (UniProt sequence P08987) and GtfD (UniProt sequence P49331) generated by the *SWISS-MODEL* web server (https://swissmodel.expasy.org) based on the catalytic domain of GtfC (PDB entry 3aie; Ito *et al.*, 2011 ▸). GtfC-CD and GtfB-CD share >90% sequence identity, while the sequence identity is between 50% and 60% for GtfD-CD. Superposition of the *SWISS-MODEL* model of GtfB-CD with chains *A* of the final structures (orthorhombic, PDB entry 8fk4; tetragonal, PDB entries 8fjc and 8fj9) in *PyMOL* shows r.m.s.d.s of only 0.3–0.4 Å. On the other hand, the r.m.s.d. values with an *AlphaFold*2 prediction were 0.5 Å. Refinement was performed using a combination of *REFMAC*5 (Kovalevskiy *et al.*, 2018 ▸; Murshudov *et al.*, 1997 ▸, 2011 ▸) in *CCP*4 (Winn *et al.*, 2011 ▸) and *Phenix *(Afonine *et al.*, 2012 ▸; Liebschner *et al.*, 2019 ▸) using local noncrystallographic symmetry (NCS) restraints. *Mogul* (CSD release) restraints for acarbose were based on the CCDC small-molecule database and were obtained from the *Grade* web server (https://grade.globalphasing.org). According to the new nomenclature rules for carbohydrate ligands (Shao *et al.*, 2021 ▸), acarbose as a ligand is split into two glucosyl monosaccharide moieties (PDB ligand ID GLC) connected to a d-saccharide containing an amino linker (PDB ligand ID AC1). *Coot* was used for all model building (Emsley & Cowtan, 2004 ▸; Emsley *et al.*, 2010 ▸) and figures were created with *PyMOL* (version 2.5.0, Schrödinger). Validations of the model quality were performed using *Phenix* and the wwPDB validation server (https://validate-rcsb-2.wwpdb.org/). The ligands were validated using the same set of *Mogul* restraints as described above. The conserved Ca^2+^ sites in the structures of the GtfB catalytic domain were verified using the *CheckMyMetal* web server (https://csgid.org/csgid/metal_sites/). Final refinement statistics are presented in Table 2 ▸.

Orthorhombic GtfB-CD in complex with acarbose (PDB entry 8fk4) crystallizes with eight molecules in the asymmetric unit, while the asymmetric unit of the tetragonal crystal forms (PDB entries 8fjc and 8fj9) contains only two molecules. The r.m.s.d. between the eight NCS-related molecules in the orthorhombic structure is 0.1 Å, while that between the two NCS-related molecules in the two tetragonal structures is slightly larger at 0.2 Å, essentially indicating high similarity.

The structures of the catalytic domains of GtfB and GtfD were deposited in the Protein Data Bank with the following IDs: 8fj9 (GtfB, apo; tetragonal), 8fjc (GtfB, acarbose complex, tetragonal), 8fk4 (GtfB, acarbose complex, ortho­rhombic), 8fkl (GtfB, apo, truncated by proteolysis, ortho­rhombic) and 8fn5 (GtfD, apo, truncated by proteolysis, orthorhombic).

### 2.5. Modeling of sucrose binding

To model sucrose binding, we used superpositions of GtfB-CD with GtfC-CD (in complex with maltose; PDB entry 3aib; Ito *et al.*, 2011 ▸) and of GTF180 in complex with maltose and sucrose (PDB entries 3kll and 3hz3, respectively; Vujičić-Žagar *et al.*, 2010 ▸).

### 2.6. Adherence of GtfB-CD to SRCR_1_ using surface plasmon resonance

We studied the adherence of GtfB-CD to SRCR_1_ by surface plasmon resonance (SPR) using a Biacore T200 instrument in the Multidisciplinary Molecular Interaction Core of the University of Alabama at Birmingham. The recombinant scavenger receptor cysteine-rich domain 1 expressed in insect cells (*i*SRCR_1_) was immobilized on a carboxymethyl dextran chip (CM5) using an amine-coupling procedure as described previously (Mieher *et al.*, 2018 ▸; Purushotham & Deivanayagam, 2013 ▸, 2014 ▸). Briefly, the chip surface was activated using *N*-hydroxysuccinimide and *N*-ethyl-*N*′-(3-diethylaminopropyl)carbodiimide, and the ligand (at 50 µg ml^−1^) in 10 m*M* sodium acetate buffer pH 4.0 was injected at a flow rate of 5 µl min^−1^ until the desired resonance units were obtained. The remaining activated group and the control were blocked by 1.0 *M* ethanolamine. After initial trial injections confirmed adherence, the analyte GtfB-CD (2–8 µ*M*) in binding buffer (20 m*M* HEPES pH 7.4, 150 m*M* NaCl, 1 m*M* CaCl_2_) was injected at 20 µl min^−1^ and the association and dissociation phases were monitored. Each concentration was tested in triplicate. The surface was prepared for the next injection using 5–10 µl injections of regeneration buffer (10 m*M* HCl). Background binding subtraction was performed by the control software. Sensorgram assessments and kinetic fittings were performed with the *Biacore Insight Evaluation* software.

## 3. Results

### 3.1. Overall structure

The extents of GtfB-CD and GtfD-CD were based on GtfC-CD, and we were able to clone, express and purify each of these constructs to obtain >95% purity based on qualitative SDS–PAGE gels. Initially, GtfB-CD, like GtfD-CD, underwent proteolysis during crystallization setups at room temperature, which we only identified after the structures had been resolved since the gels after purification appeared to be satisfactory. Our attempts to stabilize GtfB-CD with protease inhibitors proved successful. Unfortunately, use of the classic protease inhibitors AEBSF and PMSF had no effect on GtfD-CD. Through molecular replacement using homology models based on the structure of GtfC-CD and the corresponding sequences of GtfB-CD and GtfD-CD, the structures of GtfB-CD and GtfD-CD were resolved and refined.

Sequence and structural comparisons show that the catalytic domains of GtfC and GtfB are highly homologous (90% sequence identity; r.m.s.d. of 0.33 Å for 4794 of 6445 aligned atoms; see Supplementary Fig. S1). Although GtfD differs markedly in primary structure (52% sequence identity to GtfB and 61% to GtfC), it still has remarkable similarity in its structural elements (the r.m.s.d. to GtfC is 0.69 Å for 3292 of 3959 aligned atoms and the r.m.s.d. to GtfB is 0.69 Å for 3274 of 3962 aligned atoms). The active-site residues of GtfC-CD, GtfB-CD and GtfD-CD are listed in Supplementary Table S1.

GtfC-CD, GtfB-CD and GtfD-CD are structurally very similar (in secondary and tertiary structure), and this includes the catalytic residues responsible for the hydrolysis of sucrose to glucose and fructose. In addition, the residues in the adjacent Ca^2+^-binding site are also highly conserved.

**Figure 1:**
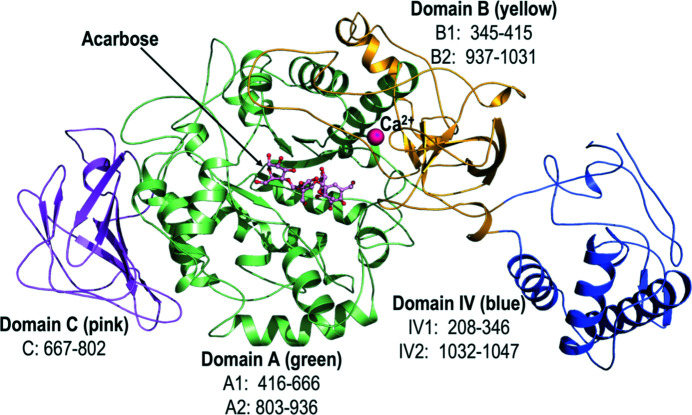
Domain structure of GtfB-CD. The figure shows the structure of GtfB-CD, which adopts a horseshoe-like arrangement. Each of the domains is colored differently and the two distinct regions that come together are indicated for domains A, B, C and IV.

Like GtfC-CD, the structures of GtfB-CD and GtfD-CD are also comprised of four separate domains: A, B, C and IV (Ito *et al.*, 2011 ▸). Among these, domain C of GtfC-CD, GtfD-CD and GtfB-CD is the only one that is built up from a contiguous single stretch, while all of the other domains are assembled from two distinctly separated regions within the linear polypeptide chain (residues from the N-terminal half and residues from the C-terminal half). The extents of the individual domains and their overall topology are shown in Table 3 ▸. Domain C is highlighted by eight β-strands in a Greek-key motif (Fig. 1 ▸), while domain A forms the core with a TIM-barrel motif like all other members of the GH13 α-amylase family, but also contains two additional helices (Supplementary Fig. S2). Domain B is formed through a twisted antiparallel sheet of six β-strands (Fig. 1 ▸) and domain IV is positioned next to domain B and could serve as a ‘hinge’ for domain V in the full-length protein (Fig. 1 ▸). Our current structure of the catalytic domain for GtfD lacks domains B1 and IV (truncated at residue 421 because of proteolysis).

### 3.2. Interaction of acarbose with GtfB and GtfC

**Figure 2:**
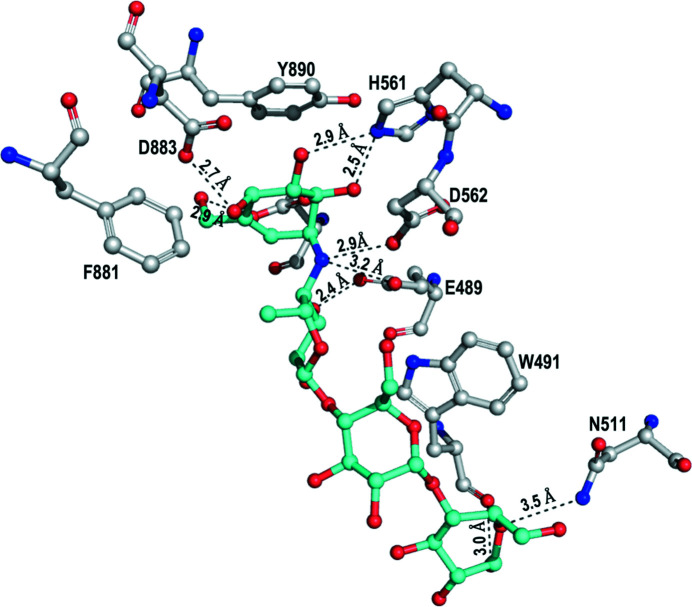
Acarbose interactions. Acarbose (cyan) and its interactions with GtfB-CD are highlighted. The hydrogen bonding is shown with distances to the various residues.

Superposition of the GtfC-CD structure (PDB entry 3aic; Ito *et al.*, 2011 ▸) with the two GtfB-CD structures (PDB entries 8fjc and 8fk4) in complex with the inhibitor acarbose (Supplementary Fig. S3) shows more than 20 conserved interacting residues in identical positions, but the ring(s) moieties of acarbose are oriented in slightly different conformations. Ito and coworkers defined subsites −1, +1, +2 and +3 based on the interactions of GtfC residues with acarbose (Supplementary Fig. S3; Ito *et al.*, 2011 ▸). In their structure, hydrogen bonds between acarbose and GtfC (PDB entry 3aic; Ito *et al.*, 2011 ▸) were observed to residues Arg475, Asp477, Asn481, Glu515, Arg540, His587, Asp588, Asp909 and Gln960. We observe corresponding conserved hydrogen bonds in GtfB to residues Arg449, Asp451, Asn455, Glu489, Arg514, His561, Asp562, Asp883 and Gln934 (see Fig. 2 ▸). However, using a hydrogen-bond distance cutoff of 3.2 Å, 12 hydrogen bonds were observed in GtfB-CD, while eight hydrogen bonds were observed in GtfC-CD. The four rings of acarbose occupy subsites −1, +1, +2 and +3. Maltose in GtfC (PDB entry 3aib; Ito *et al.*, 2011 ▸), a weak inhibitor and a transglycosyl acceptor, is bound to subsites +1 and +2, of which the glucosyl moiety interacts with active-site residues in subsite +1. Table 4 ▸ shows a *PISA* analysis (https://www.ebi.ac.uk/pdbe/pisa/) of the observed hydrogen bonds in the protein–acarbose inter­actions for the catalytic domains of GtfB and GtfC. A surface diagram colored by electrostatic potential shows that the active-site pocket is small and negatively charged (Supplementary Fig. S4).

Although the GtfB–acarbose structure in the tetragonal space group *P*4_3_22 (PDB entry 8fjc) diffracts to a higher resolution (2.50 *versus* 3.25 Å) than the GtfB–acarbose structure in the orthorhombic space group *P*2_1_2_1_2 (PDB entry 8fk4), the inhibitor acarbose fits the electron density better in the latter, possibly because of co-crystallization versus compound soaking in the former (see Supplementary Figs. S5 and S6). In the tetragonal structure (PDB entry 8fjc) the AC1 parts of the two acarbose ligands show an RSCC (based on the 2*F*_o_ − *F*_c_ density) above 0.8, while the GLC moieties are not as well refined (RSCC of 0.61–0.71). This indicates higher flexibility, since the polder omit map at the 3σ level covers more than 95% of the ligand. In the orthorhombic structure (PDB entry 8fk4) the carbohydrate moieties of the eight acarbose ligands show RSCC values in the range 0.75–0.93.

### 3.3. Modeling of sucrose into the active site of GtfB-CD and comparison with acarbose binding

The interactions with maltose in GtfC-CD (PDB entry 3aib; Ito *et al.*, 2011 ▸) were used as a model for the substrate sucrose, although the glucosyl moiety of sucrose binds in subsite −1 and the fructosyl moiety binds in subsite +1. This observation is consistent with the structures of GTF180 from *Lacto­bacillus reuteri* 180 (PDB entries 3kll and 3hz3, respectively; Vujičić-Žagar *et al.*, 2010 ▸), which highlight maltose binding in wild-type GTF180 and sucrose binding in the D1025N mutant (Vujičić-Žagar *et al.*, 2010 ▸). Binding of sucrose in comparison with acarbose for GtfB-CD is shown in Supplementary Fig. S7. Interactions of sucrose with GtfB-CD residues are listed in Supplementary Table S2.

### 3.4. Adherence to SRCR_1_

**Figure 3:**
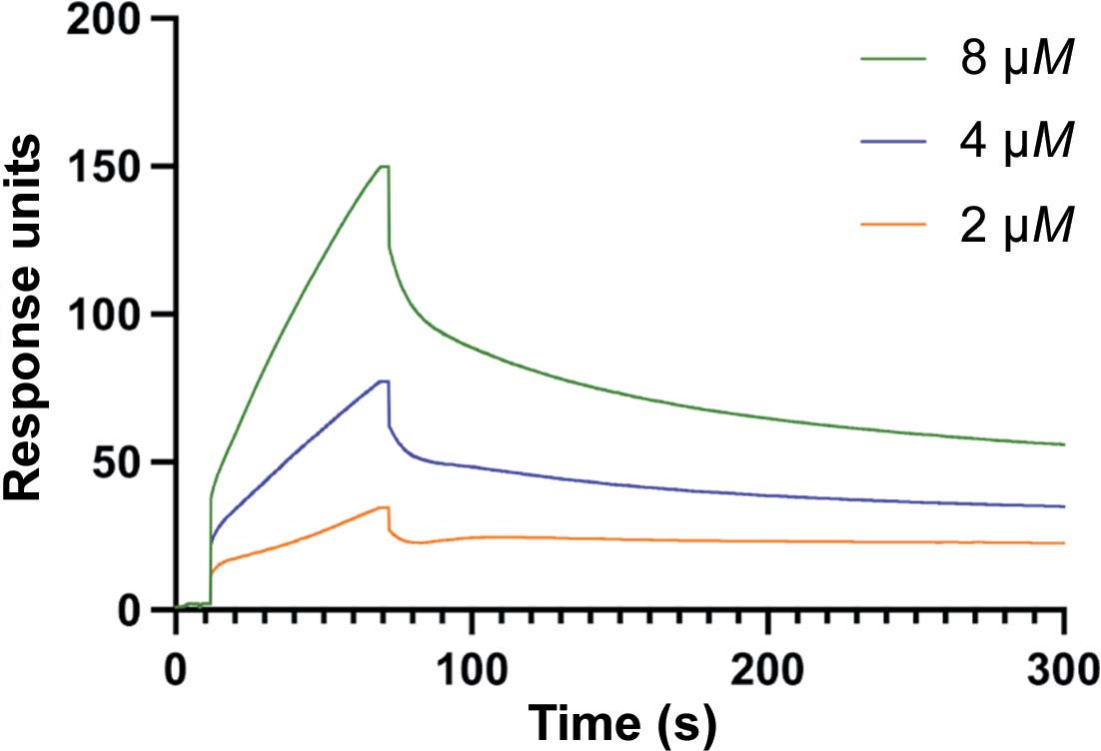
Adherence of GtfB-CD to SRCR_1_. SPR studies of the binding of GtfB-CD to SRCR_1_. The figure displays sensorgrams from SPR studies using a Biacore T200.

Increased activity of Gtfs in the presence of saliva-coated hydroxyapatite has previously been noted (Vacca-Smith & Bowen, 1998 ▸). We have also shown that a component of saliva, Gp340 (salivary agglutinin), and its scavenger receptor cysteine-rich (SRCR) domains interact with the surface proteins of *S. mutans*. We tested the adherence of GtfB-CD to SRCR_1_ in a surface plasmon resonance (SPR) experiment. The results of these studies show that GtfB-CD binds to SRCR_1_ with nanomolar affinity (*K*_d_ = 5.09 × 10^−7^ or 509 n*M*; Fig. 3 ▸; Supplementary Table S3).

## 4. Discussion

The glucosyltransferases of *S. mutans* are well known virulence factors. Their ability to convert dietary sucrose into elongated glucan polymers is an important factor in bacterial community building, and the production of acids, especially lactic acid, resulting from the metabolism of different carbohydrates wears the enamel and allows microbes to infect the tooth (dental caries). Previously, the crystal structure of the catalytic domain of GtfC was resolved, which produces both soluble (1,6-linked) and insoluble (1,3-linked) glucans. However, to date the conditions that enable such selectivity are poorly understood. In this study, we designed protein constructs for GtfB based on the catalytic domain of GtfC. The goal of our studies was to evaluate their similarities and differences and to structurally identify key residues that would play an important role in such selectivity. Toward this goal, each of these proteins was expressed and purified using multiple columns to achieve the highest purity for our structural studies.

Dental caries, which is a major health concern worldwide, is characterized by the demineralization of tooth enamel. As the main contributor to dental caries, *S. mutans* is capable of adhering to the tooth, forming a biofilm and thriving in the acidic oral microenvironment. The water-soluble and water-insoluble glucans synthesized by glucosyl transferases (GtfB, GtfC and GtfD) using sucrose as substrate play an important role in causing the irreversible attachment of *S. mutans* (and other commensal bacteria in the biofilm) to the tooth surface. Current treatment options include frequent brushing, fluoride in toothpaste, antimicrobial mouthwashes and regular dental visits. There is a need for nonbactericidal agents to selectively inhibit the cariogenic biofilms and preserve the natural oral bacterial flora.

**Figure 4:**
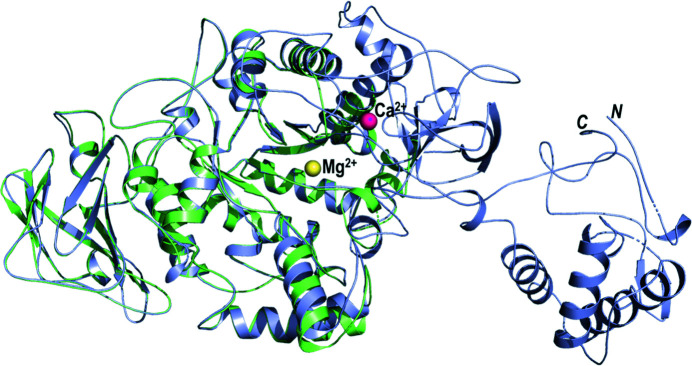
GtfB-CD apo: full-length versus truncated (proteolysed). The truncated GtfB (green) crystallized in space group *P*2_1_2_1_2_1_ and diffracted to 1.48 Å resolution. The intact GtfB-CD structure is shown in light blue. Overall there is not much difference, and the structures superpose with an r.m.s.d. of 0.25 Å (for 2702 of 3749 aligned atoms). The calcium ion is presented as a pink sphere in GtfB-CD, whereas the truncated GtfB contains a magnesium ion (yellow) at a different site.

**Figure 5:**
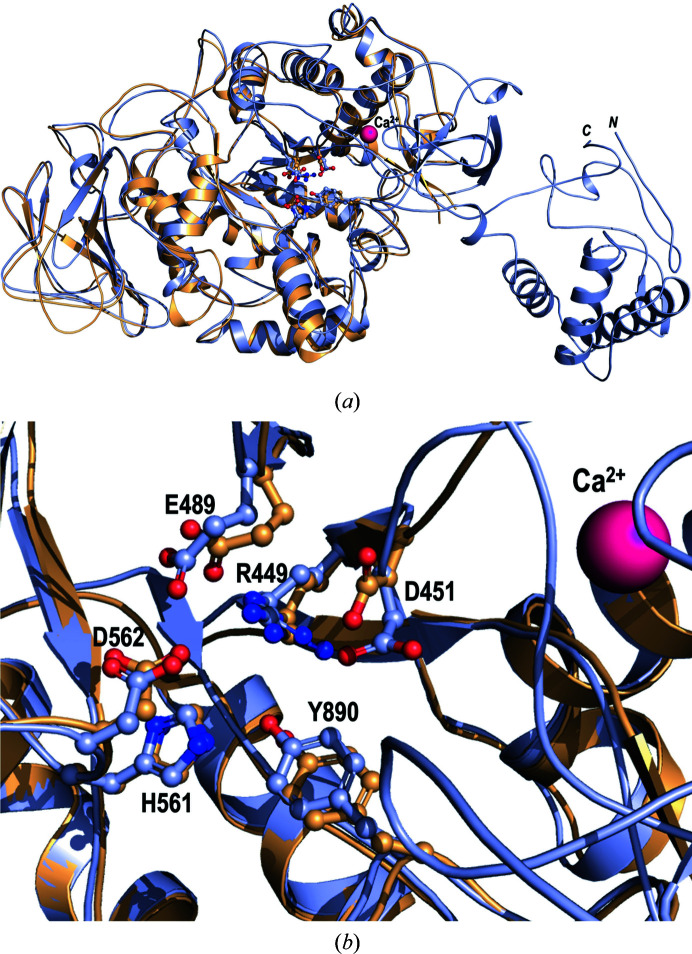
GtfD-CD (truncated) versus GtfB-CD. (*a*) The structure of truncated GtfD-CD is shown in yellow, while GtfB-CD is shown in light blue. (*b*) The conserved amino acids within the active site. The calcium ion is shown as a pink sphere.

The truncated form of GtfB-CD (completely lacking domains B and IV) crystallized in the orthorhombic space group *P*2_1_2_1_2_1_ and diffracted to 1.48 Å resolution, while the subsequently obtained apo structure of GtfB-CD in the tetragonal space group *P*4_3_22 appeared to be intact and diffracted to a resolution of 2.50 Å (for a comparison, see Fig. 4 ▸). The truncated form of GtfD-CD crystallized in the orthorhombic space group *P*2_1_2_1_2_1_ and diffracted to 1.92 Å resolution (see Fig. 5 ▸). When the models were rebuilt after obtaining molecular-replacement solutions using homology models based on the GtfC-CD structure and the GtfB or GtfD sequences, it was clear that GtfD-CD had missing residues; in fact, the final refined structure of GtfD-CD lacked domains B1 and IV. While these structures (GtfB-CD, GtfD-CD and GtfC-CD) display high structural similarity overall, there are specific differences in the residues that surround the active site. The active site for sucrose breakdown is very well conserved and highlights the same catalytic residues in GtfB, GtfC and GtfD (GtfC numbering: nucleophile Asp477, acid/base Glu515 and transition-state stabilizer Asp588; see Supplementary Table S1). Sucrose binding occurs in subsites −1 and +1, while maltose binds in subsites +1 and +2. There appears to be no room in the active site (subsites −1 and +1) for additional molecules such as glucose. An acceptor molecule or part of a growing glucan chain has to bind farther out in subsites +2 and +3, as indicated by acarbose binding, or beyond. Indeed, there is increased residue variability in these subsites, and it is postulated that domain V folds and resides close by. GtfD in particular shows several different residues, with one prominent residue (Thr589 in GtfD) being the decider for the type of glucans (soluble or insoluble) that are formed.

The enzymatic activity of the Gtfs and their dual enzymatic activity has yet to be clearly identified; namely, the hydrolysis of sucrose into glucose and fructose and the polymerization of the glucosyl moieties to form elongated glucans. Robyt and coworkers proposed an insertion mechanism in which both of the activities are contained within the same site. While these catalytic domain structures show similarities in the active site that breaks down sucrose, our analysis of the site does not provide clear evidence for such a dual enzymatic activity of Gtfs; specifically, there does not appear to be sufficient space within the active sites of GtfB and GtfD to allow the simultaneous presence of sucrose and glucose. It is our hypothesis that there must be another site in the near-vicinity of the active site of the enzyme that would carry out the polymerization step. Such a site is not clearly visible in these constructs. We therefore suggest that the N- and C-terminal regions that are not part of these structures may reveal how such a specificity to only produce soluble or insoluble glucans is embodied in each of these enzymes. It will be interesting to see how GtfC, which produces both, is regulated, and the identification of such residues could result in the development of inhibitors that would specifically target the polymerization site.

Through SPR analysis, we determined in this work that GtfB-CD adheres to the SRCR domains of Gp340. Gtfs are recognized to play a crucial role in the complex chain of events that leads to biofilm development, with the insoluble glucans that they generate sustaining the dysbiotic framework in biofilm communities. It is probable that after Gtfs are produced they attach to immobilized Gp340 on the surface of the tooth to remain in the vicinity of the bacterium. This is speculative and additional experiments would be necessary to establish this hypothesis.

In conclusion, our work demonstrates that GtfB-CD is quite similar to GtfC-CD, although their adherence to acarbose differs significantly. The modeling of sucrose in the active site demonstrates that it fits snugly into a very small pocket. The truncated model of GtfD-CD is likewise similar, but our attempts to co-crystallize acarbose with GtfD were unsuccessful due to the absence of key binding residues in the abbreviated GtfD (residues 421–1054).

## Supplementary Material

PDB reference: catalytic domain of GtfB, complex with acarbose, tetragonal, 8fjc

PDB reference: complex with acarbose, orthorhombic, 8fk4

PDB reference: apo, tetragonal, 8fj9

PDB reference: apo, truncated, orthorhombic, 8fkl

PDB reference: catalytic domain of GtfD, apo, truncated, 8fn5

Supplementary Tables and Figures. DOI: 10.1107/S2053230X23003199/jc5056sup1.pdf

## Figures and Tables

**Table 1:** Constructs for the catalytic domains of GtfB and GtfD The six nucleotides in the forward and reverse primers recognized by the listed restriction enzymes are highlighted in bold.

		Primers		
Construct	Residues	Forward	Reverse	Molecular weight (Da)
GtfB	861 (191–1051)	GGGG**CCATGG**GGGATGAAACTGGCGCTTATACT (NcoI)	GAA**CTCGAG**TTTATTATCTGAAATATTAAA (XhoI)	96330
GtfD	854 (248–1091)	GCTC**GCTAGC**ATTGATAACTATGTCACAGCTGATTC (NheI)	GCTC**CTCGAG**GTTAGTCATCTGTTTTGGCAGAT (XhoI)	95663

**Table 2:** Crystallographic parameters

Structure	GtfB-CD apo (truncated)	GtfB-CD + acarbose	GtfB-CD + acarbose	GtfB-CD apo	GtfD-CD (truncated)
PDB code	8fkl	8fk4	8fjc	8fj9	8fn5
Data collection					
Space group	*P*2_1_2_1_2_1_	*P*2_1_2_1_2	*P*4_3_22	*P*4_3_22	*P*2_1_2_1_2_1_
*a*, *b*, *c* (Å)	86.67, 90.86, 92.26	299.31, 215.77, 219.33	149.55, 149.55, 303.09	150.13, 150.13, 302.78	60.63, 126.77, 173.36
Resolution (Å)	1.48	3.25	2.50	2.50	1.92
Unique reflections	121563 (5970)	222519 (10910)	119150 (5822)	119867 (5852)	97783 (4973)
Completeness (%)	99.9 (100)	99.9 (100)	100 (100)	100 (99.8)	95.6 (98.5)
Multiplicity	7.4 (7.2)	6.8 (7.1)	10.2 (10.8)	7.4 (7.2)	3.6 (3.7)
*R*_merge_ (%)	8.1 (62.7)	18.4 (241.4)	25.2 (183.7)	31.4 (396.6)	10.0 (79.7)
*R*_p.i.m._ (%)	3.2 (25.5)	7.6 (97.2)	8.4 (59.5)	7.2 (88.2)	5.7 (46.9)
CC_1/2_, highest shell	0.861	0.394	0.446	0.439	0.657
〈*I*/σ(*I*)〉	13.6 (2.8)	7.4 (1.0)	9.3 (1.8)	9.3 (1.1)	7.7 (1.7)
Wilson *B* (Å^2^)	19.6	99.3	41.2	51.3	25.9
Refinement					
Resolution (Å)	1.48	3.25	2.50	2.50	1.92
Unique reflections	121512 (12032)	222189 (7357)	119032 (3915)	119738 (8700)	97717 (9919)
Completeness (%)	99.8 (100)	99.8 (100)	99.9 (100)	100 (100)	95.1 (98.1)
*R*_work_ (%)	14.9 (18.5)	22.4 (37.7)	18.6 (26.7)	21.2 (33.7)	22.2 (35.1)
*R*_free_ (%)	17.0 (21.2)	25.5 (40.4)	22.8 (34.0)	24.1 (35.4)	24.2 (35.5)
Average *B* (Å^2^)					
All	30.0	103.8	45.8	59.7	33.5
Protein	28.8	103.6	44.8	59.8	33.6
Waters	40.3	61.4	41.7	48.2	30.8
R.m.s.d., bond lengths (Å)	0.027	0.020	0.011	0.013	0.013
R.m.s.d., angles (°)	1.68	0.56	1.21	1.71	1.71
CC (*F*_o_ − *F*_c_)	0.97	0.94	0.94	0.94	0.95
Ramachandran statistics					
Favored	98%	92.8%	97.0%	96%	95.8%
Outliers	2	0.4%	1	1	0.4%
Clashscore	2.29	4.83	3.65	3.90	5.19
*MolProbity* score	1.01	1.71	1.33	1.42	1.57

**Table 3:** Domain assignment for the catalytic domains of GtfB, GtfC and GtfD

	A1	A2	B1	B2	C	IV1	IV2
GtfB (GTF-I)	417–666	803–936	347–416	937–1031	667–802	220–346	1032–1062
GtfC (GTF-SI)	443–692	829–961	372–442	962–1056	693–828	246–371	1057–1087
GtfD (GTF-S)	431–688	834–967	360–430	968–1062	689–833	233–359	1063–1091

**Table 4:** *PISA* analysis of acarbose (ACR) structures (hydrogen bonds) The distance cutoff for hydrogen bonds used here is 3.2 Å. For both structures chain *A* was used in the comparison (GtfC, PDB entry 3aib; GtfB, PDB entry 8fjc).

GtfC				GtfB			
Residue	Atom	ACR atom	Distance (Å)	Distance (Å)	ACR atom	Atom	Residue
Arg475	NH2	O2A	3.05	3.05	O2A	NH2	Arg449
Asp477	OD2	O6A	3.23	2.83	O6A	OD2	Asp451
Asn481	ND2	O2B	2.50	3.02	O2B	OD1	Asn455
Glu515	OE2	N4B	3.13	2.80	O3B	OE1	Glu489
				2.39	O1D	ND2	Asn511
Arg540	NH2	O1D	3.03	3.17	O6D	NH2	Arg514
Arg540	NH1	O1D	3.01				
His587	NE2	O3A	2.28	2.83	O3A	NE2	His561
				2.80	O2A	NE2	His561
Asp588	OD2	N4B	3.19	3.09	N4B	OD2	Asp562
				2.83	O2A	OD2	Asp562
				2.67	O4A	OD2	Asp883
				2.77	O6A	NE2	Gln934
