# Supplementary material for: The catalytic domains of Streptococcus mutans glucosyltransferases: a structural analysis
Source: Acta Crystallogr F Struct Biol Commun. 2023 May 5;79(Pt 5):119–27. doi: 10.1107/S2053230X23003199 (PMC10167749; doi:10.1107/S2053230X23003199)
Supplement: Supplementary file 1 [file f-79-00119-sup1.pdf]

Acta Cryst F

STRUCTURAL BIOLOGY COMMUNICATIONS

Volume 79 (2023)

Supporting information for article:

The catalytic domains of *Streptococcus mutans* glucosyltransferases: a structural analysis

Norbert Schormann, Manisha Patel, Luke Thannickal, Sangeetha Purushotham, Ren Wu, Joshua Mieher, Hui Wu and Champion Deivanayagam

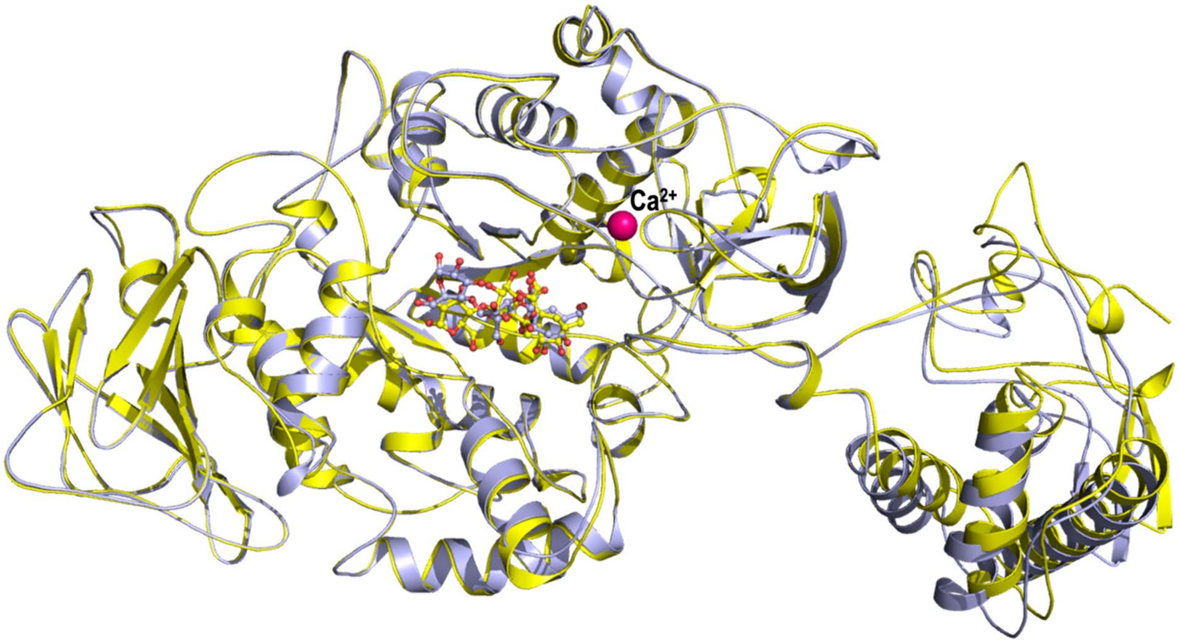

Figure S1 GtfC versus GtfB. Superposition of GtfB-CD shown in light blue (this study) and GtfC-CD (3AIC) shown in yellow. Acarbose binding to the active site is also shown.

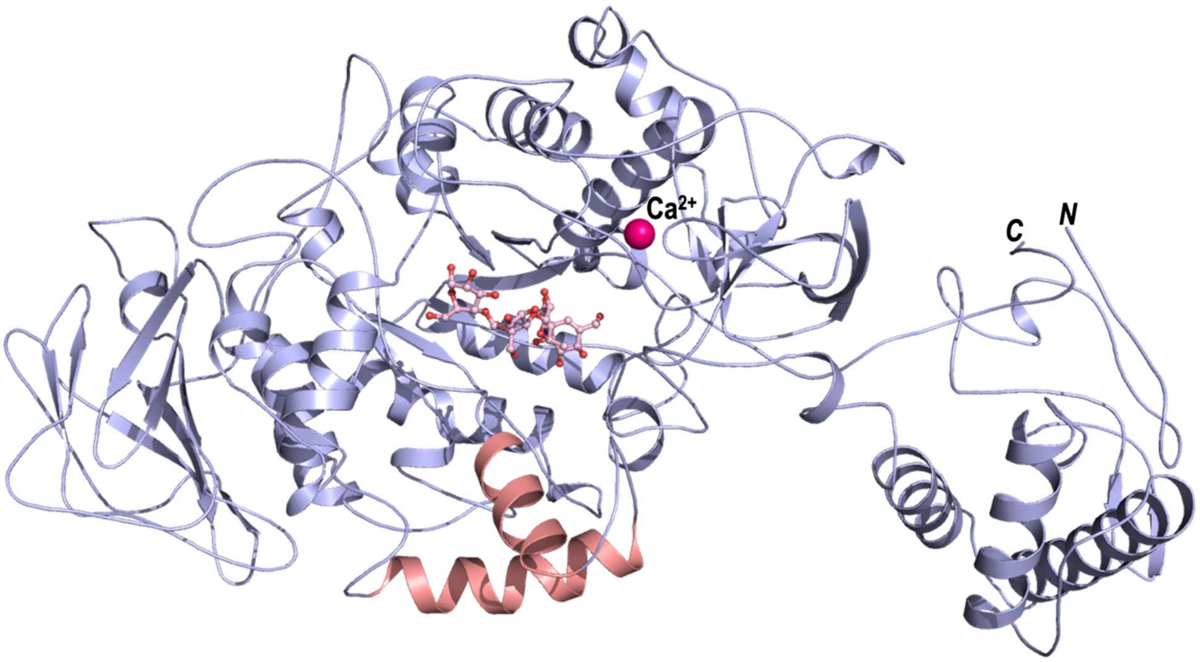

Figure S2 Extra Helices in Domain A of GtfB-CD. GtfB-CD is shown in light blue, and the two additional helices present on GtfB are shown in salmon. These residues span GtfB's α4' helix are residues 566-577 and α4" residues 588-603.

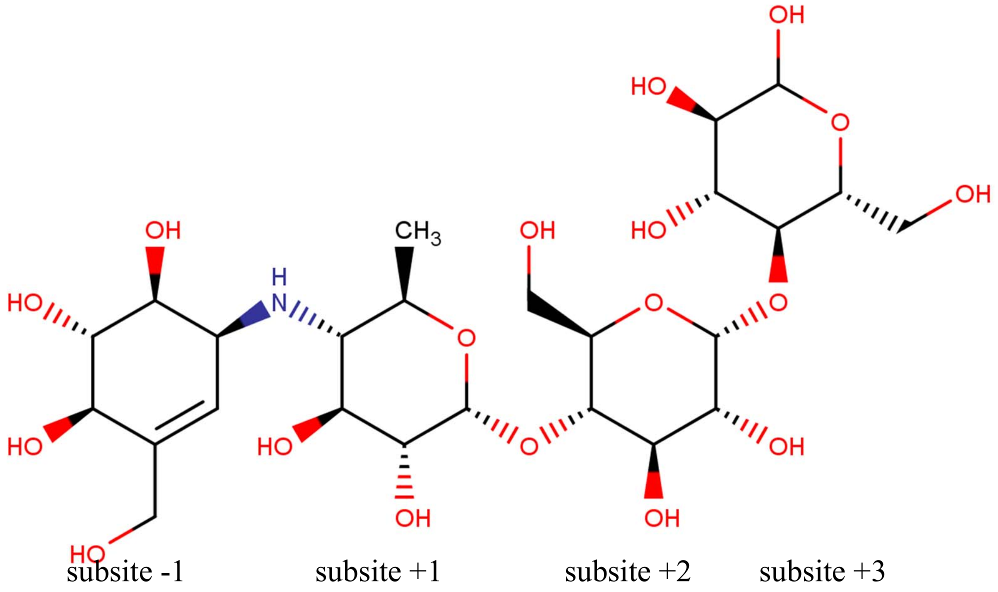

Figure S3 2D structure of acarbose and assignment to subsites in GtfB-CD based on crystal structures. 4,6-dideoxy-4-{[(1S,4R,5S,6S)-4,5,6-trihydroxy-3-(hydroxymethyl)cyclohex-2-en-1-yl]amino}-alpha-D-glucopyranose-(1-4)-alpha-D-glucopyranose-(1-4)-alpha-D-glucopyranose (AC1-GLC-GLC).

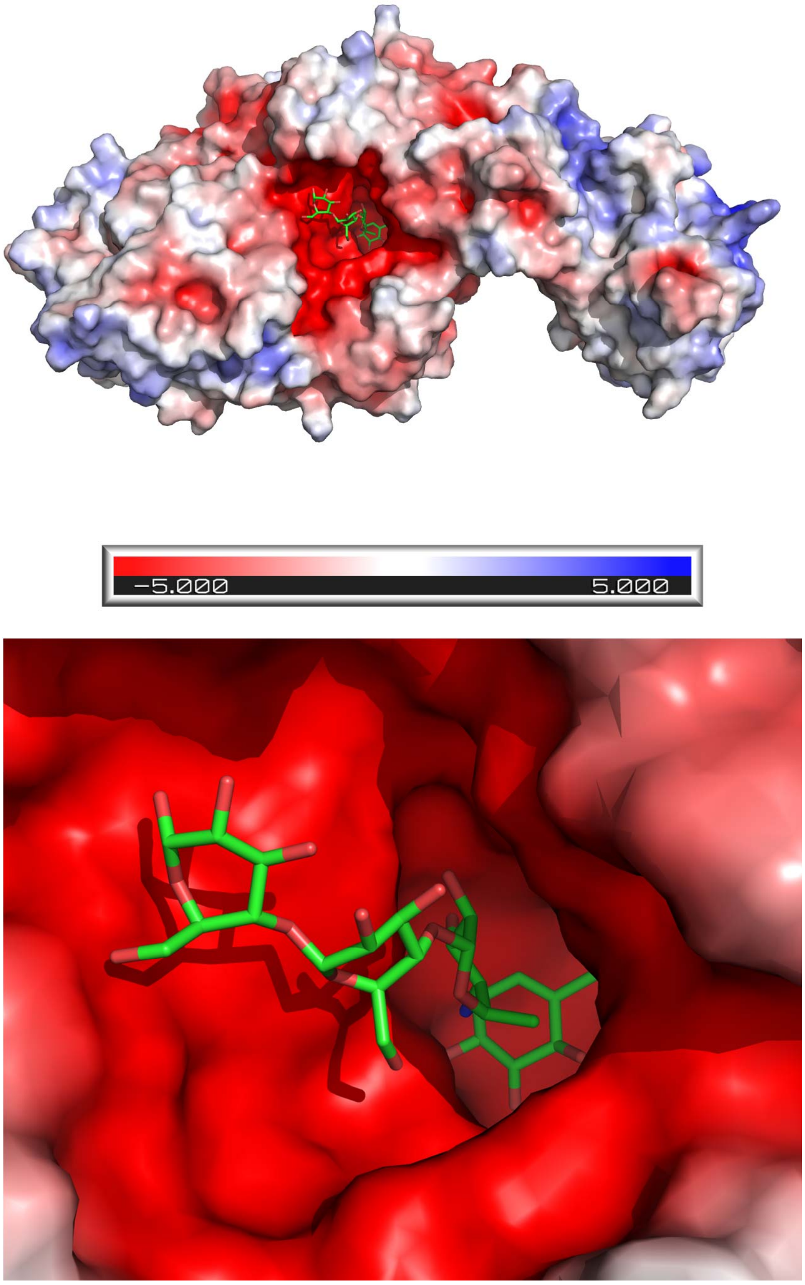

Figure S4 GtfB-CD in complex with acarbose in surface representation colored by electrostatic potential. Surface diagram colored by electrostatic potential for GtfB-CD (entire catalytic domain and close-up of pocket), where acarbose is shown in a green stick model. Contours for the electrostatic potential are -5*kT* in red and +5*kT* in blue.

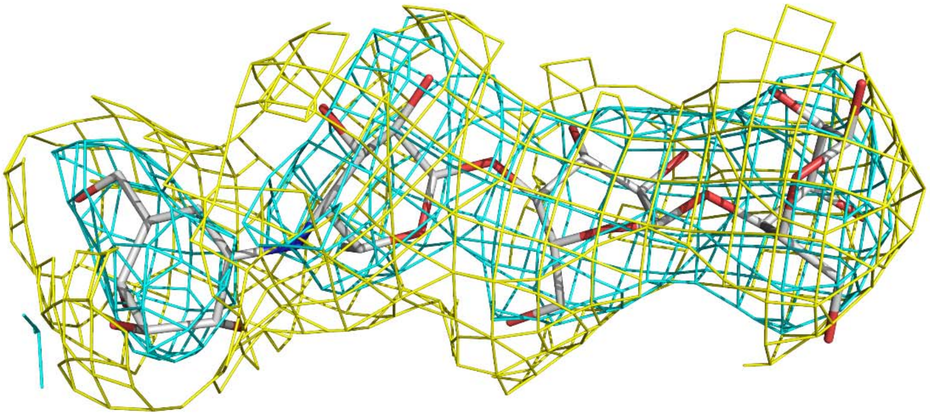

Figure S5 Map quality of acarbose in chain A of orthorhombic GtfB-CD (PDB8FK4). Cyan: 2Fo-Fc map at 1σ contour leve. Yellow: Fo-Fc polder omit map at 3σ contour level

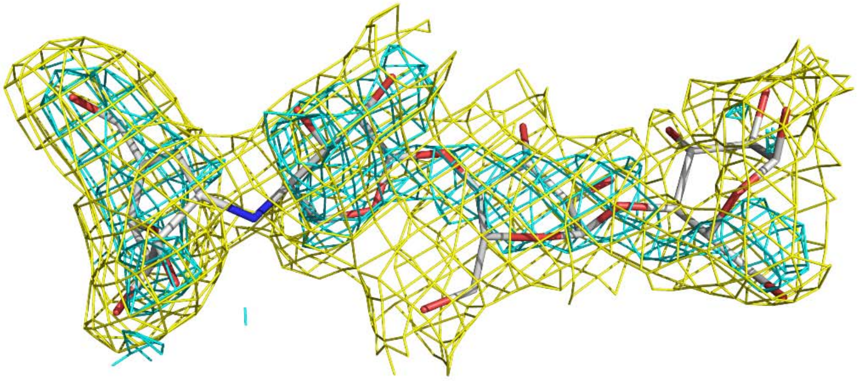

Figure S6 Figure S6. Map quality of acarbose in chain A of tetrameric GtfB-CD (PDB 8FJC). Cyan: 2Fo-Fc map at 1σ contour level. Yellow: Fo-Fc polder omit map at 3σ contour level

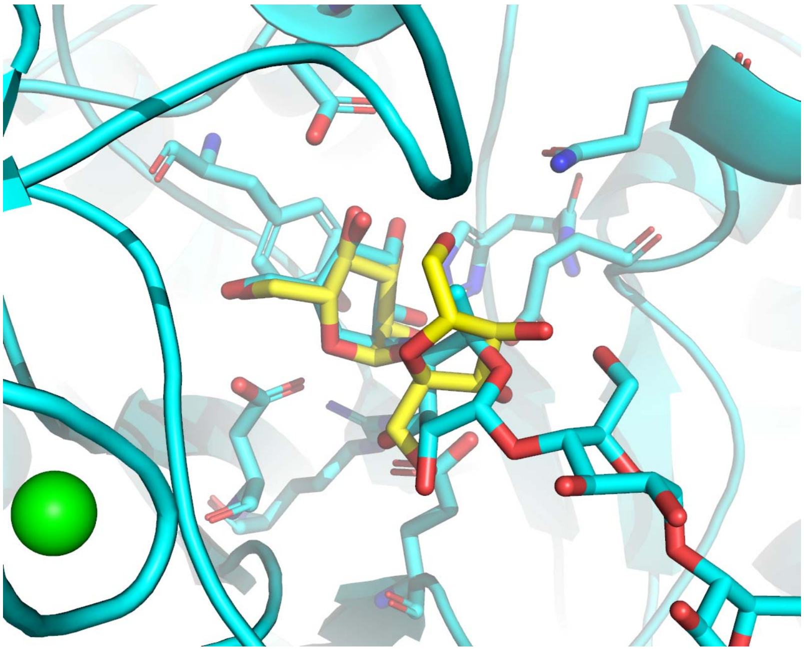

Figure S7 Sucrose modeling and comparison with acarbose binding.

Table S1 Active site and potential polymerization site residues in GtfB-CD, GtfC-CD and GtfD-CD

GtfB-CD	GtfC-CD	GtfD-CD
Asp451, Glu489, Asp562	Asp477, Glu515, Asp588	Asp465, Glu503, Asp584
Lys523, Asp564, His611	Lys549, Asp593, His637	Arg537, Thr589, Gln633

Second row: Active site residues.

Third row: Potential polymerization site residues.

Table S2 Interactions of modelled sucrose with GtfB-CD residues

A. Hydrogen bonds

GtfB Residue	GtfB Atom	Distance [Å]	SUC Atom
**Arg449**	**NH2**	**3.30**	**O2**
**Asp451**	**OD2**	**2.95**	**O6**
**Trp491**	**NE1**	**3.27**	**O3’**
**His561**	**NE2**	**2.91**	**O3**
**Gln934**	**NE2**	**2.93**	**O6**

The distance cutoff for hydrogen bonds here is 3.3Å. Note that atoms in the fructosyl moiety are numbered O6’, C6’, C5’, C4’, O4’, C3’, O3’, C2’, O2’, C1’, O1’ while atoms in the glucosyl moiety are labelled O6, C6, C5, O5, C4, O4, C3, O3, C2, O2, C1, O1.

B. Hydrophobic interactions

SUC interface residues
**Leu356, Leu407, Leu408, Ala452, Asn455, Glu469, Asp562, Tyr584, Asn836, Phe881, Asp883, Asn888, Tyr890**

Table S3 Experimental parameters for the GtfB-CD adherence to SRCR1

Analyte	Ligand	k_a (1/Ms)[a]	k_d (1/s)[b]	K_D (1/M)[c]	R_{max} (RU)[d]	Chi² (RU²)
GtfB-CD	SRCR1	2.99E+03	1.52E-03	5.09E-07	117.20	86.9

[a] k_a, association rate constant.

[b] k_d, dissociation rate constant.

[c] K_D, equilibrium dissociation constant.

[d] R_{max}, maximum analyte binding capacity. RU, response units.
